# Prevalence of food allergy in prescholl children

**DOI:** 10.1186/1939-4551-8-S1-A198

**Published:** 2015-04-08

**Authors:** Laura Carneiro Matoso Nunes Canabrava, Marcela Rezende, Ana Carolina Nápolis, Rodrigues Silva Segundo Segundo, Tássia Cecília Pereira Guimarães,

**Affiliations:** 1 Universidade Federal De Uberlandia, Brazil

## Background

The objective of this study was to know the prevalence of food allergy in preschool children enrolled in municipal daycare centers from Uberlândia, Minas Gerais, Brazil.

## Methods

This is an observational study that enrolled children from 24 to 59 months in municipal daycare centers from Uberlândia. A self-administered questionnaire that was used to evaluate the prevalence of food allergy related by parents; subsequently, children with a suspecting food allergy were invited to a clinical and laboratory evaluation, in order to know the real prevalence of food allergy.

## Results

13,841 children enrolled, 8,031 parents responded the questionnaire. The prevalence of food allergy reported by parents was 17.6% and the main foods mentioned were cow milk, pork, fruit, chocolate and chicken egg, and associated symptoms were red spots (54.2%), vomit (39.6%), diarrhea (32.1%), abdominal pain (31.4%), mouth and eyes edema (17.5%) and nose secretion (10.6%). After clinical and lab evaluation, the prevalece of food allergy found was 0.59% of preschool children, which 0.35% was IgE -mediated and 0.24% was non- IgE mediated reactions. The egg was the main food allergen, reaching 0.34% of preschool children, followed by cow milk (0.21%), wheat and pork meat (0.06%), corn, mustard, honey and fish (0.03%). The main symptoms were red spots and itching (52.6%), diarrhea (42%), eyes edema and abdominal pain (36.8%).

## Conclusions

The prevalence of food allergy in preschool children found was inferior than described previously. There is a great difference in the parental perception and the real prevalence of food allergy. Several patients was undergoing an inadequate diet exclusion, that results in a reduction of quality of life and could impact in the nutritional status.

